# Therapeutic efficacy of fixed dose artesunate-mefloquine for the treatment of acute, uncomplicated *Plasmodium falciparum* malaria in Kampong Speu, Cambodia

**DOI:** 10.1186/1475-2875-12-343

**Published:** 2013-09-23

**Authors:** Rithea Leang, Sakun Ros, Socheat Duong, Visweswaran Navaratnam, Pharath Lim, Frédéric Ariey, Jean-René Kiechel, Didier Ménard, Walter RJ Taylor

**Affiliations:** 1 National Centre for Parasitology, Entomology and Malaria Control, #372, Monivong Blvd, Corner St. 322, Phnom Penh, Cambodia; 2 Drugs for Neglected Diseases initiative, Pulau Pinang, Malaysia; 3 Institut Pasteur du Cambodge, Phnom Penh, Cambodia; 4 Drugs for Neglected Diseases initiative, Geneva, Switzerland; 5 Centre de Médecine Humanitaire, Hôpitaux Universitaires de Genève, Geneva, Switzerland

**Keywords:** *Malaria*, *Plasmodium falciparum*, Cambodia, Artesunate, Mefloquine, Drug resistance

## Abstract

**Background:**

Cambodia stopped using co-blistered, non-fixed, artesunate-mefloquine (ASMQ) in 2008 when treatment failure rates approximated 20%. Fixed dose combination (FDC) ASMQ is efficacious against acute uncomplicated, drug resistant *Plasmodium falciparum* malaria in Southeast Asia but has not been tested in Cambodia.

**Methods:**

A 42-day WHO therapeutic efficacy study (TES) was conducted in 2010 in Oral, Kampong Speu province, south-west Cambodia, in patients with acute uncomplicated *P*. *falciparum*. Daily administered FDC ASMQ for three days was dosed by age. Genotyping of isolates at day 0 and day of recrudescence by polymerase chain reaction (PCR) classified post-treatment recurrent falciparum parasitaemia. *Ex vivo* drug sensitivity testing ([^3^H] hypoxanthine method) was performed on baseline parasites and reported as the drug concentration inhibiting 50% parasite growth *vs* no drug (IC_50_).

**Results:**

Recruited patients numbered 45; five aged <15 years. On day 3, five of 45 [11.1 (3.7-24.05)] % patients were still parasite-positive; one of whom later failed treatment on day 21. There were 5/45 (11.1%) late treatment failures on day 21, 28 and 35; all were PCR diagnosed recrudescent infections. The day 0 MQ IC_50_s ranged from 11.5-238.9 (median 58.6) nM.

**Conclusions:**

This TES demonstrated reasonable efficacy in an area of possible reduced artemisinin sensitivity and high MQ IC_50_s. Efficacy testing of FDC ASMQ should continue in Cambodia and be considered for reintroduction if efficacy returns.

## Background

The first artemisinin-based combination therapy (ACT) to be introduced in 2000 by the National Malaria Control Programme in Cambodia (CNM, Centre National de Malariologie) to treat drug-resistant *Plasmodium falciparum* was non-fixed artesunate-mefloquine (NF-ASMQ). Prior to this, there had been a long history of anti-malarial drug resistance, starting with chloroquine [1] in the early 1960s, followed by sulphadoxine-pyrimethamine (SP) [2], mefloquine (MQ) [3] and SP combined with MQ [4].

NF-ASMQ was co-blistered (i e, both drugs in the same blister). There were five age-weight dosing categories and the maximum MQ dose was capped at 1,500 mg because of its poor tolerability [5]. When dosed by age rather than weight, a substantial minority of patients (~30% of all patients, ~43% adults) received less than the total target doses of AS and MQ over 48 hours, i e, <12 mg/kg of AS and <20 mg/kg of MQ. Nevertheless, between 2001 to 2004, therapeutic efficacy studies (TESs), conducted across Cambodia, demonstrated generally PCR-corrected rates of adequate clinical and parasitological response (ACPR) exceeding 90% when assessed over 28 days [5,6].

However, as early as 2001, the day 28 ACPR rate in Pailin was low at 85.7% and was about the same, 90.1%, in 2004. A subsequent, day 42 follow-up TES in 2004 in Pailin demonstrated an ACPR of just under 80% [5]. Analyses of some of the falciparum parasites from these TESs [6] showed a significantly lower geometric mean MQ IC_50_ (drug concentration required to inhibit parasite growth by 50% compared to a no-drug control) in parasites with 1 *Pfmdr*-*1* copy number compared with parasites with ≥2 copies (27.9 nM *vs* 50.3 nM) and a lower median *Pfmdr*-*1* copy number in patients with ACPR compared those who failed treatment: 1.5 *vs* 2.4 [7]. The risk of failing ASMQ was about eight-fold more likely with ≥3 *Pfmdr*-*1* copy numbers [7].

NF-ASMQ TESs conducted in 2007 in Veal Veng (Pursat province, western Cambodia) and in 2006–08 in Chumkiri (Kampot province, south-western Cambodia) demonstrated day 42 PCR-corrected failure rates of 16% and just under 19%, respectively [8,9]. Independent risk factors for treatment failure in Kampot were a high day 0 parasite density, longer time to clear parasites and an increasing *Pfmdr*-*1* copy number [9].

*In vitro* drug sensitivity data from Chumkiri showed that the MQ IC_50_s ranged from approximately 15 to 180 nM ([^3^H] hypoxanthine method) and a higher mean IC_50_ was reported in resistant infections (90 nM) *vs* sensitive (56 nM) infections [9]. These high IC_50_ values are consistent with *in vitro* data from Lim *et al*. who reported a geometric mean IC_50_ of 50.57 nM in MQ failures *vs* 23.85 nM in ACPR patients from west and east Cambodia combined in studies from 2001 to 2007 [10].

Based on these high failure rates and the earlier poor efficacy of artemether-lumefantrine [6], ASMQ was replaced by fixed dose dihydroartemisinin-piperaquine (DHA-PP) in 2008 because of its convenient daily dosing and high efficacy in Cambodia [11] and elsewhere in southeast Asia [12].

Recent DHA-PP efficacy data from western Cambodia, on relatively small patient numbers, have been discouraging. PCR-corrected failure rates of 25 and 10% have been reported from Pailin and Pursat, respectively; both areas have high rates (19 and 27%, respectively) of pfmdr1 positive falciparum parasites and *in vitro* data characterized by high IC_50_s for MQ [13]. Western Cambodia has well documented artemisinin resistance (AR), manifest as slow asexual parasite clearance and a consequential increase in gametocytogenesis [14]; thus, ACT failures may be due to AR, partner drug resistance and poor drug absorption or a combination of all three.

FDC-ASMQ, a comparatively new ACT, has been WHO prequalified [15] but has never been tested in Cambodia. If its efficacy were high enough, it could be recommended by CNM. Herein, the results of a TES using the fixed dose ASMQ in Cambodia are reported.

## Methods

### Study design and site

**Figure 1:**
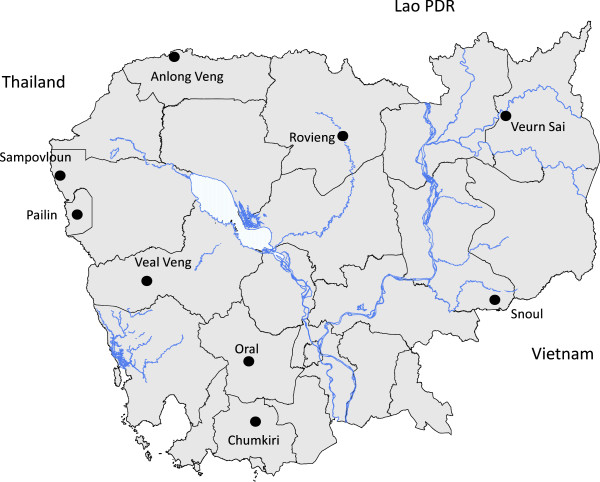
Location of the study sites, Oral, Kampong Speu province, Cambodia.

This standard WHO TES [16] was conducted in Oral Health Centre in Oral district, Kampong Speu province, about 100 km south-west of Phnom Penh (Figure 1). Malaria is seasonal with an approximate *Plasmodium falciparum*: *Plasmodium vivax* ratio of 2:1. The high transmission season is from June to November [17]. The study took place from September 2010 to January 2011. *In vitro* data (P Lim, unpublished observations but aggregated in reference [10]) from this area in 2001 (n = 13 isolates) and 2003 (n = 54) show median (interquartile ranges, [full ranges]) IC_50_s for MQ of 17.3 nM (14.6-36.1) [11.9-69.9] and 8.4 nM (3.6-27.5) [1–143.4], respectively.

The study was approved by the National Ethic Committee for Health Research of the Ministry of Health, Cambodia.

### Patients

Patients who attended the Oral Health Centre were enrolled if they met all of the following inclusion criteria: (i) informed consent was signed; (ii) they had a documented fever or a history of fever within the previous 48 hours; (iii) microscopic *P*. *falciparum* between 1,000 and 200,000 asexual parasites/μL; and, (iv) were aged two years and above.

Exclusion criteria included: (i) pregnant or breast feeding women; (ii) unable to complete the follow-up; (iii) allergic to AS or MQ; (iv) a neuropsychiatric contraindication to MQ, namely, epilepsy, history of a severe psychiatric illness, e g, bipolar affective disorder, psychosis, anxiety neurosis; (v) presence of any danger signs or any sign of signs of severe malaria [18]; (vi) severe malnutrition; and, (vii) known severe underlying disease.

### Study conduct

Patients who were malaria slide-positive were asked if they were interested to join a research study. After informed consent was signed, they were assessed to see if they met the criteria for study enrolment. This involved a medical history, physical and blood examinations for a repeat malaria slide (Giemsa-stained and read in the field), haematocrit, total white cell count and blood glucose.

Parasite counts were determined on Giemsa-stained thick films and recorded as the number of parasites per 200 white blood cells. Two qualified microscopists read independently all malaria slides and parasite densities were recorded as the average of these two counts. Slides were to be reread if the parasitaemia difference was >50%. The parasitaemia/μL was determined by multiplying the parasite count/200 white cells by 40. A slide was declared negative after reading 100 thick film fields.

Filter-paper blood spots were collected on day 0 and on the day of recurrent parasitaemia to determine if the recurrence was a resistant (recrudescent) or new infection by the polymerase chain reaction (PCR) amplification of the genes of the merozoite surface protein 1 and 2 (*msp 1*, *msp 2*) and the glutamate rich protein (*glurp*) [19].

Patients were followed up for 42 days. They were seen daily for temperature measurement, asked about symptoms and had repeat malaria slides until two consecutive malaria slides were negative. Thereafter, they were seen weekly from days 7 to 42. Home visits were done if patients missed their appointments.

### Study drug

ASMQ, supplied by Farmanguinhos in Brazil, was administered under supervision in the clinic, following the manufacturer’s instructions. Each higher dose tablet contained 100 mg artesunate and 220 mg mefloquine hydrochloride (200 mg base) and each lower dose tablet 25 and 55 mg, respectively. The doses were: (i) adults (≥13 years) - two high-dose tablets per day for three days; (ii) older children, seven to 12 years, one high-dose tablet per day for three days; and, (iii) younger children one to six years, two lower-dose tablets per day for three days.

If vomiting occurred within 30 minutes, a full dose (two tablets) was to be re-administered; if between 31 and 60 minutes, one tablet. All treatments were given supervised by study nurses. Other drugs were allowed to be given as clinically indicated, e. g, paracetamol for fever and an antiemetic for nausea and vomiting. Drugs with anti-malarial activity, e g, certain antibiotics were not given unless necessary.

### Outcomes

Efficacy outcomes were based on the WHO TES classifications: adequate clinical and parasitological response (ACPR), late parasitological failure (LPF), late clinical failure (LCF) and early treatment failure (ETF) [20].

### Primary endpoint

The primary endpoint was day 42, PCR-corrected, Kaplan Meier (KM) proportions of patients with an ACPR.

### Secondary endpoints

The secondary efficacy endpoints were: (i) the proportions of patients with positive parasites on day 3; (ii) parasite clearance time (PCT): the time in days for two successive malaria slides to become and remain negative; (iii) fever clearance time (FCT): time to become afebrile for at least 24 hours; and, (iv) gametocyte carriage (proportion of patients gametocyte positive) over time.

### Definitions of treatment failure

An ETF was defined as: (i) the development of severe malaria within the first 72 hours; (ii) a day 2 parasite count > day 0 parasite count; (iii) a day 3: day 0 parasite count ≥25%; and, (iv) parasitaemia on day 3 with fever. A LTF was an initial clearing of parasites by day 7 followed by a recurrence of falciparum parasitaemias without fever. A LCF was a LTF with a fever.

In accordance with WHO recommendations, treatment failure was labelled recrudescence if all *msp*-*1*, *msp*-*2*, and *glurp* alleles present at the time of failure had been present at the time of treatment initiation [21]. In all other cases, the failure was considered a new infection.

### Treatment emergent symptoms and signs

A treatment emergent symptom and sign (TESS) was defined as being absent pre-FDC-ASMQ but reported or detected post-drug administration. TESSs were sought only during the first three days and were not graded by severity. A serious adverse event (AE) was an AE with at least one of the following characteristics: (i) life-threatening; (ii) resulted in death; (iii) caused residual significant disability; or, (iv) resulted in a prolongation of a patient’s hospital stay [22].

### *Ex vivo* drug sensitivity testing

*Ex vivo* drug sensitivity testing was performed at the Institut Pasteur du Cambodge (IPC) using the 48 hours [^3^H] hypoxanthine isotopic method [23], the same method used in earlier studies [10]. Samples were only tested if they arrived within 48 hours of being taken, the patient had not taken anti-malarial drugs and the parasitaemia was between ~0.1 to 1%.

Briefly, artesunate, chloroquine, dihydroartemisinin, mefloquine, and quinine were obtained from Sigma-Aldrich (Singapore) and piperaquine from Yick-Vic Chemicals & Pharmaceuticals (Hong Kong). Stock solutions of anti-malarial drugs were prepared in 0.5% lactic acid for piperaquine, and methanol for the other drugs. The final plate concentrations ranged from 0.1-102.4 nM for artesunate, 5–5,120 nM for chloroquine, 0.0625-64 nM for dihydroartemisinin, 1–1,024 nM for mefloquine, 2–2,000 nM for piperaquine, and 6.25- 6,400 nM for quinine. Each concentration was used to coat two wells in a 96-well flat-bottom plate (ATGC, France). Forty μl of drug solutions were added to each well. Plates were dried into a laminar flow hood (per batch of 30 plates) and kept at 4°C until they were used. Batches of pre-dosed plates were prepared weekly and generally used within two weeks after preparation. Their suitability for *in vitro* testing was monitored regularly using the reference *P*. *falciparum* strain 3D7 maintained in continuous culture. The results of the *in vitro* assay were expressed as the 50% inhibitory concentration (IC_50_), defined as the concentration at which 50% of the incorporation of [^3^H] hypoxanthine was inhibited, compared to the drug-free control wells. These IC_50_s are determined by non-linear regression using the ICEstimator [24].

### Data management and statistical analysis

The sample size was calculated based on standard WHO criteria and used the precision method [20]. With a confidence of 95%, an assumed failure rate of 15% with 10% precision (i e, from 5 to 25%), the estimated sample size was 50 evaluable patients. In accordance with WHO TES, Kaplan Meier (KM) analysis was used to assess the day 42 cure rate [20]. Data were collected onto a standard case record form (CRF) and double entered in the WHO Excel spreadsheet. Stata version 10.1 (Stata Corp, College Station, TX, USA) was used for statistical analysis. Patients were included in the KM analysis if they received at least one dose of FDC-ASMQ. Patients without a parasitological endpoint, e g, lost to follow-up, were censored in the KM analysis.

Continuous data were summarized using mean, standard deviation (SD), median, interquartile (IQR) and full ranges and compared by *t*-test (normally distributed data) or the Mann Whitney *U* tests (skewed data). Simple proportions were compared using chi squared. P-values of ≤0.05 were considered statistically significant.

### Role of the funding agency

The Drugs for Neglected Diseases *initiative* (DND*i*) funded the study, monitored the fieldwork and contributed to this publication.

## Results

Fifty patients were screened, five did not meet the entry criteria and 45 were enrolled. The majority of patients were adults of median age 22 years; 38 patients were males (Table 1). Patient flow in the study was characterized by no losses to follow-up and five patients with recurrent parasitaemia.

**Table 1:** Enrolment characteristics of the 45 study patients

**Parameter**	**Value**
Males*	38 (84.44)
Females*	7 (15.56)
Age years†	22 (7–56)
Age 2–15 years*	5 (11.11)
Adults*	40 (88.89)
Weight in kg†	50 (18–67)
Temperature in °C†	38.5 (36.8-40.8)
Febrile (≥37.5°C)*	39 (86.67)
Parasitaemia in N/μl†	15,778 (10,215-24,369)
Gametocyte positive*	0 (0)
Haematocrit % †	38 (34–43)
Artesunate dose in mg/kg in 48 h	12 (10.9-13.9, 8.9-17.1)
Mefloquine dose in mg/kg in 48 h	24 (21.8-27.9, 17.9-34.3)

* N (%); † Median (range); Median (interquartile range, range).

The median total daily doses over 48 hours of AS and MQ were 12 and 24 mg/kg, respectively (Table 1). Two patients aged <13 years were inadvertently given two FDC tablets per day instead of one. Excluding these two, the median doses received were 11.8 and 23.5 mg/kg, respectively. Had the patients received NF ASMQ, as recommended by the May 2012 National Treatment Guidelines, median (range) doses would have been 11.8 (8.3-15) mg/kg for AS and 22 (12.5-28.1) mg/kg. The mean difference in the MQ dose between the FDC and NF ASMQ (excluding the two given the wrong dose) was 1.9 mg/kg (p < 0.0001).

## Outcomes

### Primary efficacy endpoint

There were no cases of ETF. By day 42, there were five LCFs which occurred on days 21 (n = 1), 28 (n = 3) and 35 (n = 1). All were classed as recrudescences for a KM cure rate of 88.9 (75.3-95.2) %. The median total doses of AS and MQ in the recrudescent infections were essentially the same as the cured patients: 12 *vs* 11.9 and 23.7 *vs* 24 mg/kg, respectively.

### Day 3 positivity

On day 3 (72 hours), five of 45 [11.1 (3.7-24.05)] % patients were still parasite-positive; one of whom later failed treatment on day 21. Their day 0 day 3 parasite counts (N/μL) were: 13,067-270, 14,074-953, 30,718-1872, 46083–434, and 108,013-134.

### Parasite and fever clearance times

The PCT ranged from two to seven days for a median of three days. All febrile patients at presentation (n = 39) were afebrile by day 2 for a median FCT of 24 hours.

### Gametocyte carriage

Gametocytes were not detected on days 0 to 21 and 35 and 42; 4/44 (9.1%) patients had gametocytes on day 28.

### TESS

ASMQ was well tolerated acutely. There were no patients with drug-induced vomiting who needed rescue treatment. Over the first three days, seven patients reported adverse events, five of whom had received >24 mg/kg of mefloquine. Two (4.4%) patients complained of dizziness, five (11.1%) complained of nausea, and five (11.1%) complained of palpitations. Of these, one complained of all three symptoms and three complained of nausea and palpitations. None required treatment.

### *Ex vivo* drug sensitivity testing

Among the 45 isolates collected, 18 met the criteria for *ex vivo* testing. These 18 patients had similar baseline characteristics and received similar drug doses as the 27 who did not undergo *ex vivo* testing. Excluding the untested patients with parasite counts ≤ 5,000/μL, the median baseline parasite counts were not significantly different similar (p = 0.37): 39,815 (n = 18) vs. 20,103/μL (n = 19). The median IC_50_s (including IQR and range) are presented in Table 2. The variation in IC_50_ values varied from four-fold (piperaquine) to approximately 60-fold (CQ). Of the 16 ASMQ treated patients with IC_50_ data, only one was classed as a treatment failure: 55.3 nM. The IC_50_ values in patients with ACPR ranged 11.5 to 238.92 for a median of 58.74 nM. The results were almost the same when stratifying by day 3 positivity.

**Table 2:** **IC**_**50**_**results obtained from the*in vitro*drug sensitivity tests**

**Drug**		**IC**_**50**_**nM**			
	**N**	**Median**	**IQ range**	**Range**	**Fold difference**
Mefloquine	16	58.6	42.8-152.9	11.5-238.9	20.8
Artesunate	18	1.9	1.7-2.3	0.45-4.9	10.9
Dihydroartemisinin	18	2.1	1.1-2.8	0.18-5.2	28.9
Piperaquine	13	46.5	36.1-48.1	26.5-104.7	4.0
Chloroquine	16	206.2	146-267	4.9-301.9	61.6
Quinine	13	365.8	235.5-638	132-724.8	5.5

Using WHO suggested [25]*in vitro* IC_50_ cut off values for distinguishing resistant from sensitive parasites (30 nM for mefloquine, 400 nM quinine, and 100 nM for CQ): (i) 14/16 MQ tested parasites had evidence of *in vitro* resistance: median (range) IC_50_ = 65 nM (35–239); (ii) 5/13 (quinine) IC_50_ = 666 nM (603–725); and, (iii) 13/16 (CQ) IC_50_ = 209 nM (138–302).

## Discussion

This TES has shown that the FDC-ASMQ was reasonably effective in an area of south-west Cambodia, achieving a day 42 cure rate a little under 90%, in the face of *in vitro* data suggesting reduced MQ sensitivity and where the day 3 positivity rate exceeded the original 3% cut-off [26] for suggesting reduced artemisinin sensitivity as well as the 10% cut-off adapted by WHO for malaria control programmes [25].

NF-ASMQ was used on a national scale from 2000 until 2008 when it was replaced by DHA-PP after failure rates of some ~20% were reported from Pailin and Kampot [8]. Thus, this FDC-ASMQ TES study was done after an interval of approximately two years since NF-ASMQ was replaced in Cambodia.

The FDC-ASMQ doses administered to the patients were almost identical for AS but the mean MQ dose was slightly higher compared to the Cambodian, NF-ASMQ dosing recommendations. Although about half of the patients received doses under the targeted doses of AS and MQ, none received <6 mg/kg of AS, the suggested therapeutic minimum [27], and none received <15 mg/kg of MQ, a minimum suggested by malariologists. The failure rate was 11% but given the small sample size the confidence interval is wide, ranging from <5 to <25%. Therefore, caution is warranted in interpreting this result even though it appears better than failure rates reported earlier from Pailin and Kampot.

The MQ *in vitro* data in this study were characterized by a high median IC_50_, ~three- to seven-fold higher compared to data obtained in 2001 and 2003 in Oral, respectively, and is comparable to geometric mean *in vitro* values associated with NF-ASMQ treatment failures in some studies [10] but is lower than the median IC_50_ found by Rogers *et al*. in Kampot in resistant infections (90 nM) [9]. As expected, the relatively high day 3 positivity rate and the *in vitro* data appear to have had little predictive value for ASMQ failure.

Malaria control programmes should be aware that the day 3 positivity rate is an indicator of the possibility that parasites may be artemisinin resistant [28] and that this should trigger a more detailed *in vivo* investigation in which the parasite clearance half-life should be measured. The current *in vitro* methods are useful to track trends over time but are insensitive for detecting artemisinin resistance. However, a new *in vitro* test, the RSA (Ring-stage Survival Assay) [29], is showing promise in correlating with slow parasite clearance in patients [30].

The study had limitations. The number of patients enrolled in the TES was quite small, 45, due to the low malaria burden and this reduces the strength of the study conclusions. Most patients recruited were adult males, consistent with the malaria epidemiology in other areas of Cambodia. Follow-up extended to day 42 so the true failure rate may have been a little higher had follow-up extended to 63 days, the recommended follow-up time for long half drugs such as mefloquine [31]. These limit the ability to interpret and apply the results. The *Pfmdr*-*1* copy number was not counted. This molecular marker of reduced MQ sensitivity [32] is another good marker of the trends in MQ sensitivity. Indeed, in Pailin, the *Pfmdr*-*1* copy number declined markedly from 33% in 2005 to 5% in 2007 [33]. The WHO TES places little emphasis on the collection of tolerability data. Thus, the tolerability data reported here focused only on new, post-treatment TESS. This was a limitation but the toxicity of MQ and NF-ASMQ is well characterized.

To conclude, this TES has benchmarked in the *in vivo* and *in vitro* status of FDC-ASMQ in Oral. The 11% failure rate is not discouraging and CNM should continue to monitor FDC-ASMQ efficacy, preferably to day 63, in conjunction with *in vitro* data and *Pfmdr*-*1* copy number to see if FDC-ASMQ can be recommended in the future for treating symptomatic patients. FDC-ASMQ should also be assessed in PCR positive asymptomatic individuals for a potential role in malaria elimination.

## Competing interests

The authors have declared that there are no competing interests.

## Authors’ contributions

The study PI was LR. LR and JRK adapted the WHO TES protocol. LR, RS and DS performed the study. *In vitro* drug sensitivity testing was done by DM and previous *in vitro* data were given by PL and FA. The study was coordinated and monitored by VN and JRK. WRJT, RL and DM analysed the data and wrote the first draft of the paper. All authors have seen and approved the final manuscript.
